# The Marriage of Syphilitics*The Surgical Section of the College of Physicians of Philadelphia.

**Published:** 1894-07

**Authors:** William G. Porter

**Affiliations:** Senior Surgeon to the Philadelphia Hospital; Surgeon to the Presbyterian Hospital, Philadelphia, Pa.


### THE MARRIAGE OF SYPHILITICS.*

By WILLIAM G. PORTER, M.D.,

Senior Surgeon to the Philadelphia Hospital; Surgeon to the Presbyterian Hospital, Philadelphia, Pa.

Perhaps no more important or interesting question could have been submitted for our discussion to-night than that of the Marriage of Syphilitics, and I must confess that I approach its consideration with an amount of diffidence which I would not have supposed to be possible some years ago. It would be very easy to dispose of the whole question by giving to our patients who have

*The Surgical Section of the College of Physicians of Philadelphia.

been afflicted with syphilis and who are contemplating matrimony the celebrated advice of Mr. Punch to those about to be married, "*Don't!*"

But in regard to this question we have a solemn duty to perform; on our decision may depend the future happiness and health, not only of our patient, but that of an innocent and virtuous woman and of children yet unborn.

The subject is so large in all its bearings that it will be simply impossible for me to more than outline some of its more salient points to-night. In approaching the subject we must consider first *our patient.*

If we forbid him to marry, and he takes our advice, we condemn him to perpetual celibacy—we rob him of all the joys of matrimony, of possible paternity.

If we are right in our judgment we have nothing with which to reproach ourselves.

But if we have acted inadvisedly; if he is really incapable of transmitting the disease to others; if he is capable of procreating healthy children; if he has a long life of health and usefulness before him; if, in other words, he is cured, we have done him a lasting injury; we have blighted his life; we have robbed the State of a citizen who would in all probability have been more useful married than single.

On the other hand, if we advise our patient, who is or has been syphilitic, that he can safely marry, and he follows our advice and infects his wife or procreates syphilitic children, we have not only injured him, but inflicted irreparable injury on an innocent wife and helpless children.

Now, gentlemen, are there any rules which may guide us in the determination of this most important question? Of course, in an assemblage of physicians like this, it would be entirely out of place for me to address you as I would a class of students. You are all more or less familiar with the disease. Many of you are experts in its detection and treatment, both as specialists and as general practitioners. In the limited time at our disposal it would be impossible to offer the proofs of all the statements I shall make; I must therefore endeavor to limit myself to propositions which, I

think, we can all indorse, and see if we can reasonably make any deductions from them.

In the first place, then, I would say that syphilis may be classified for our purpose into three varieties, viz., the benignant, the moderate, the malignant.

I suppose that it has occurred to all of us *occasionally* to have seen cases of primary syphilis which have been followed by an exceedingly moderate amount of secondary symptoms, and then, almost without treatment, by perfect recovery and the absence of further manifestations; to these I would apply the name of benignant.

By moderate syphilis I mean those ordinary cases in which the disease runs through its various stages without severe symptoms—perfectly responsive to treatment, generally controlled by it, but which it may take many months or perhaps some years to cure.

By malignant syphilis I mean those cases which are severe from the start, in which the earliest secondary symptom may be a rupia with a profound cachexia, or immediately on the disappearance of the chancre, or even sometimes before it has disappeared, the nervous system of the patient may be profoundly affected.

Recognizing these three varieties, my next proposition is that all three forms are generally curable: the first exhausting itself, the latter two requiring the treatment of the *skillful* physician to overcome them. Gentlemen, I say *skillful* physician, for there is no disease which responds more rapidly to proper treatment or shows worse results when improperly treated than syphilis.

We know that syphilis is curable from the fact that many patients remain indefinitely without a recurrence of symptoms, and more than all that, they not infrequently contract it a second time. But up to the present time no means has been discovered by which we can say absolutely to any individual patient, "You are cured; you will never have a return of this disease unless you contract it again." Because of this many writers on syphilis claim that as we can never say absolutely that a patient is cured, we have no moral right to advise him to take the chances of matrimony; that we can never tell, even after the lapse of years, when a relapse may occur, and the patient, who has supposed himself to be cured, may transmit to his posterity this terrible disease. Now, it seems to me if there is one thing established in medicine, it is the power of medical treatment over the manifestations of syphilis. Who among you has not seen symptoms disappear under treatment almost as if by magic, cases apparently the most hopeless restored to health, and yet it happens to all of us occasionally to see cases which, in spite of systematic and long-continued treatment, persistently relapse. They are not really ill; they go about attending to business and the affairs of life as usual; and yet we know and they know that they are not well; the slightest indiscretion is sure to produce a temporary relapse, and so it goes on for years and years. I think that all of us who have been in practice for twenty-five years or more must recognize the fact that we do not see nearly as many severe cases of syphilis as we formerly did, and yet if we consider the numberless pathological conditions which it most assuredly causes, we must admit that it is still a most serious affection, and one which results much more frequently in death than is generally supposed.

Now, under what, if any, circumstances, are we justified in informing a patient who has suffered from this disease that it will be safe for him to marry?

In the first place, marriage should be absolutely forbidden to any patient who presents at the time he consults us any of the symptoms of syphilis—primary, secondary, or tertiary. Let me quote here from the master on this subject—Fournier:

"What then is marriage in its completeness, gentlemen? Marriage is not only an affair of sentiment, of passion, of convenience, and of interest; to consider it from a standpoint more practical and at the same time more elevated, marriage is an association freely entered into, where each contracting party is pledged to bring in good faith a share of health and physical vigor, with the view of co-operating, on the one hand, for the material prosperity of the family, and, on the other hand, for the raising of children, the supreme and sacred end of every union.

"Now what, in this case, I ask you, will be the share contributed to the partnership by a husband syphilitic, and not cured of his syphilis? His share will be that of a health compromised, hypothecated, burdened with a debt hereafter due the pox, that pitiless creditor.

"On account of the pox it may happen that this man may experience one day or another such and such serious affections which will ruin his health, such and such an infirmity which will render him incapable of work, incapable of earning his daily bread; and then what will become of the family, of which this man is the recognized support? What will become of his wife? What will become of his children?

"On account of the pox also this man may die. What may happen, he being dead, to his wife and to these children?

"Is it admissible, then, that a man should think of creating for himself a family when he is liable to fail this family? Is it admissible? Is it right? Is it moral that a man should dream of having a wife and children when he offers the possible prospect of widowhood to this wife, of orphanage to these children, of poverty to this family? No, a hundred times no!

"Also, and I do not hesitate to say it, the man who is syphilitic, and not cured of his syphilis, fears not nevertheless to append his signature to the marriage contract, commits at this moment a base act, an act immoral and corrupt, an act which good people will be unanimous in condemning."

In the second place, marriage should be absolutely forbidden to all syphilitic patients who have not been subjected to a most thorough, complete, and prolonged treatment.

And finally, in the third place, before sanctioning the marriage of a syphilitic, the requirement of a sufficient treatment having been fulfilled, the physician should be satisfied that he is in perfect health, and that he has had no symptoms, even suspicious, of syphilis for a period of at least two years.

Under these circumstances, and with these limitations, I have no hesitation in telling patients that I think they are reasonably safe in marrying, and I have never yet had reason to regret the advice which I have given.

